# Hints for the Treatment of Suddenly-Developed Insanity

**Published:** 1898-06-18

**Authors:** F. St. John Bullen

**Affiliations:** Late Assistant


### Medical Progress and Hospital Clinics.

[*The Editor will be glad to receive offers of co-operation and contributions from members of the profession. All letters should be addressed to* The Editor, at the Office, 28 & 29, Southampton Street, Strand, London, W.C.]

### HINTS FOR THE TREATMENT OF SUDDENLY-DEVELOPED INSANITY.

By F. St. John Bullen, M.R.C.S., Late Assistant Medical Officer and Pathologist, West Riding Asylum.

There are few emergencies more trying to the practitioner, particularly if he has not had special training in mental diseases, than the urgent summons to treat a person who has suddenly developed symptoms of acute mental disorder. On such an occasion the patient's friends are usually so helpless, through their mingled dread and ignorance of the condition, and their entire inability to cope with it, that the medical man has to assume control of all arrangements needful to restore order to a disorganised household. It is therefore desirable that he should be prepared to deal with insanities of peculiarly sudden onset needing immediate treatment, and in some cases the transient duration of these may make it advisable to attempt this at home. Some hints may now be offered as to temporary proceedings for the safe guarding of the patient and his friends, and as to the method of summary sequestration.

Although it is seldom that mental derangement has an absolutely sudden onset, yet, so far as the medical man is concerned, this might often be considered the case, inasmuch as the patient's friends have perhaps not observed, or have temporised with, the warning symptoms, and have only sought help when these have become urgent. The most rapidly-developed form of mental disorder is known as transitory insanity or frenzy, a very active delirium with much motor agitation. No satisfactory explanation as to the cause, direct or indirect, of this rather uncommon outbreak may be found.

The frenzy can occur uncomplicated, or may come on in connection with the parturient period at or shortly after delivery, with an epileptic discharge, general paralysis, alcoholic poisoning, dipsomania, brain hæmorrhage, shock, &c. Less sudden in origin, of longer duration, and without the intensely rapid delirium of frenzy, are states of delirious mania and melancholia which, like transitory madness, may develop with or without complications. As regards emergency treatment, it is not out of place to group them with the last-mentioned.

**Ephemeral Insanity,** as its name implies, runs a speedy course, and has a cessation almost as abrupt as its invasion. A period of twelve hours usually covers the attack, but an interval of quiescence may occur earlier, to be followed by another outburst, or the attack may even be prolonged over two days. As a rule, the more rapid the onset and acute the excitement the sooner the termination, and the less effectual the use of therapeutic measures, so that the attack will probably subside before removal of the patient to an institution can be undertaken; and hence in these frenzies the object must be to protect the patient and his surroundings from the result of his delirium. Complete preparations for the efficient management in a private dwelling of such suddenly aroused excitement will seldom be feasible, but the room to be occupied by the patient should be immediately cleared of all superfluous furniture, breakable articles, and those possibly constituting lethal weapons, whilst, unless there be reason to expect a very transient attack, the windows should be guarded.

The patient is best allowed free movement unless his freedom be obviously attended with danger to himself or his surroundings; if kept in bed, this should be composed of mattrasses stretched upon the floor. Although the principal safeguard is the assistance of reliable attendants, yet from the possible difficulty in procuring these, as well as from the short period during which the frenzy lasts, other procedures must be considered, such as restraint by chemical or mechanical means. The psychosis in question is notably intractable to the influence of drugs; so furious is the mental tumult that short of an almost lethal dose of some paralysing agent, such as hyoscyamine, no quieting effect within the limited duration of the attack can usually be expected. In the case of a patient to be treated in a private dwelling, and too dangerous to be allowed freedom of movement, reliance will have mainly to be placed upon restraint of mechanical kind. A ready and good mode of applying this consists in the wet pack, especially as this not only limits movement, but has some therapeutic value. Essential to its application are several blankets, a sheet, and tepid water, all easily procured. Upon a mattrass placed on the floor is spread a piece of waterproofing, when obtainable, on that two or three blankets, an old one uppermost; lastly, a sheet soaked in the water, and not too thoroughly wrung out. Upon the damp sheet the patient is placed recumbent, with arms stretched by his side, and in it he is to be enveloped; the various blankets are then successively folded round with their edges alternately placed. In a hour's time, if no symptoms of exhaustion have ensued, the patient should be unpacked, and after being briskly rubbed with a rough towel the pack should be re-applied. If he be robust the process of packing may be extended over a period of 12 hours, the same care always being exercised.

When the application of the pack is impracticable the writer would advise the following mode of procedure: The patient, being held recumbent on a mattrass, a sheet, folded lengthwise until about a foot wide, is wrapped round one knee, carried across the other knee, around both until firmly bound, and then secured with safety-pins; the sheet must be wrapped around one knee first to avoid chafing. The arms can be well secured thus: Carry a roller-towel across the back of patient's shoulder, pass the arms through the loop-ends, and twist the towel with a stick—tourniquet fashion—over the spine until the arms are fixed backwards and to the side of the chest. A figure-of-eight band will prevent the stick from untwisting; a cushion must be placed under the head. The chest is left sufficiently free for breathing purposes, and thus trammelled the patient can be easily controlled for a considerable length of time.

(*To be continued.*)

